# Application of the optimizing health literacy and access (Ophelia) process in partnership with a refugee community in Australia: Study protocol

**DOI:** 10.3389/fpubh.2023.1112538

**Published:** 2023-02-21

**Authors:** Zaman Jawahar, Shandell Elmer, Melanie Hawkins, Richard H. Osborne

**Affiliations:** Centre for Global Health and Equity, Swinburne University of Technology, Melbourne, VIC, Australia

**Keywords:** health literacy development, co-design, former refugee community, Ophelia process, Conversational Health Literacy Assessment Tool (CHAT)

## Abstract

Refugees experience health inequities resulting from multiple barriers and difficulties in accessing and engaging with services. A health literacy development approach can be used to understand health literacy strengths, needs, and preferences to build equitable access to services and information. This protocol details an adaptation of the Ophelia (Optimizing Health Literacy and Access) process to ensure authentic engagement of all stakeholders to generate culturally appropriate, needed, wanted and implementable multisectoral solutions among a former refugee community in Melbourne, Australia. The Health Literacy Questionnaire (HLQ), widely applied around the world in different population groups, including refugees, is usually the quantitative needs assessment tool of the Ophelia process. This protocol outlines an approach tailored to the context, literacy, and health literacy needs of former refugees. This project will engage a refugee settlement agency and a former refugee community (Karen people origin from Myanmar also formerly knowns as Burma) in codesign from inception. A needs assessment will identify health literacy strengths, needs, and preferences, basic demographic data and service engagement of the Karen community. This community will be engaged and interviewed using a semi-structured interview based on the Conversational Health Literacy and Assessment Tool (CHAT) will cover supportive professional and personal relationships, health behaviors, access to health information, use of health services, and health promotion barriers and support. Using the needs assessment data, vignettes portraying typical individuals from this community will be developed. Stakeholders will be invited to participate in ideas generation and prioritization workshops for in-depth discussion on what works well and not well for the community. Contextually and culturally appropriate and meaningful action ideas will be co-designed to respond to identified health literacy strengths, needs, and preferences of the community. This protocol will develop and test new and improved methods that are likely to be useful for community-based organizations and health services to systematically understand and improve communication, services and outcomes among disadvantaged groups, particularly migrants and refugees.

## 1. Introduction

The complexity of health issues arising from the movement of refugees around the world poses challenges for health systems. Former refugee groups often experience challenges in accessing and using healthcare services because of economic and legal limitations, language barriers, lack of knowledge about their health rights, and other socio-cultural, administrative, and institutional barriers (1–3). Consequently, former refugee communities often tend to be overlooked by mainstream health programs and so do not receive fit-for-purpose health education and information. These systemic barriers result in delayed or no access to health services (4). Each year, ~4,000 refugees settle in the Australian State of Victoria through the humanitarian program (5). In Victoria, as with many regions around the world, the size of refugee populations is increasing, leading to the need for improved processes for health services to understand and respond to their health issues.

Globally, there is a limited research on the health issues faced by refugees after they have resettled in a destination country, including Australia (6–10). Refugees have relatively poor health and encounter barriers to accessing healthcare services. These barriers include the lack of culturally and linguistically sensitive health services and information, which cause difficulties in navigating the health system and for understanding or interpreting health information (11–13). The pre-migration, migration and resettlement experiences of refugees have multiple impacts on their health and wellbeing. These impacts vary across individuals, families, and communities depending on country of origin, duration of the migration experience, and their pre-existing health behaviors and ailments (14). Previous traumatic experiences of refugees have a persistent impact on their health and wellbeing after arrival in host countries (15, 16). Refugees may have experienced interruptions in access to healthcare services in their country of origin due to war and conflict (17). Consequently, they may have inadequately managed diseases, injuries, as well as have ongoing mental health issues due to trauma (18–20). In addition to experiencing conflict, poverty and variable access to health services, the settlement process in the host countries may aggravate health inequities and increase exposure to various health risks. The factors leading to the poor health of refugees in destination countries are well documented (21–23). However, a deeper understanding is required to inform health and community services about ways to better respond to these identified needs (24).

Researchers, public health practitioners and service providers across the health system attempt to identify and eliminate disparities in the health and wellbeing of former refugees. However, it is challenging to design approaches that examine underlying causes and worldviews that influence cultural beliefs, norms, values, health behaviors, and expectations (25). While a focus on these has raised awareness of the needs of these communities, much of the work to date has focused on deficits that refugees might have (e.g., what they can't do or are lacking in) (26). This has led to stereotyping such refugees as “hard to reach” (27, 28). A deficit approach, with a predilection for identifying weaknesses and problems limits research processes to capture potential strengths such as community values, resilience, tacit knowledge, skills and competencies that may be used to build a more comprehensive understanding of a population and inform the development of wholistic solutions.

Health literacy is a multidimensional concept that has recently evolved to be a valuable problem-solving tool to assess and understand both the strengths and challenges of individuals and communities including those who do not access services (26, 29–31). According to the WHO, health literacy represents “the personal knowledge and competencies that accumulate through daily activities and social interactions and across generations. Personal knowledge and competencies are mediated by the organizational structures and availability of resources that enable people to access, understand, appraise and use information and services in ways that promote and maintain good health and wellbeing for themselves and those around them” (31, 32). Different groups of people may have different sets of health literacy strengths, needs and preferences. This has important implications for understanding what is really required to determine how to build services and initiatives that may help different communities, especially those who come from diverse cultures, including refugees (26, 33, 34).

Health literacy initiatives are particularly important for refugees to facilitate uptake of available health services and information (35). Few studies provide insights into health literacy initiatives suitable for refugee populations (36–39). The limited research suggests that needs assessments, using community participatory approaches and plain language are useful (40). However, there are very few studies about methods for identifying and addressing the health literacy needs in the refugee setting. A systematic review of randomized control trials was conducted to identify methods and outcomes that aimed to improve health literacy and behaviors of refugee communities (41). Overall, the studies in the field of refugees' health literacy were highly heterogenous in terms of study groups (e.g., immigrants, refugees, and asylum seekers) (see Table 1 for migration terminology), research design, metrics, methods, and overall methodologies and did not critically assess the health literacy needs and local knowledge of refugee groups (41).

**Table 1:** Migration terminology.

**Terminology**	**Definition**
Pre-Migration	“Pre-migration is the stage in the relocation process when the refugees are in their home countries and are deciding and preparing to move to a safe country” (42).
Migration	“Migration is the process of social change whereby an individual moves from one cultural setting to another for the purposes of settling down either permanently or for a prolonged period” (43).
Resettlement	“Resettlement is the transfer of refugees from an asylum country to another State, that has agreed to admit them and ultimately grant them permanent residence” (44).
Immigrants	“From the perspective of the country of arrival, a person who moves into a country other than that of his or her nationality or usual residence, so that the country of destination effectively becomes his or her new country of usual residence” (45).
Refugee	“A person who qualifies for the protection of the United Nations provided by the High Commissioner for Refugees (UNHCR), in accordance with UNHCR's Statute and, notably, subsequent General Assembly's resolutions clarifying the scope of UNHCR's competency, regardless of whether or not he or she is in a country that is a party to the 1951 Convention or the 1967 Protocol – or a relevant regional refugee instrument – or whether or not he or she has been recognized by his or her host country as a refugee under either of these instruments” (45).
Asylum Seeker	“An individual who is seeking international protection. In countries with individualized procedures, an asylum seeker is someone whose claim has not yet been finally decided on by the country in which he or she has submitted it. Not every asylum seeker will ultimately be recognized as a refugee, but every recognized refugee is initially an asylum seeker” (45).
Former Refugee	A former refugee means an individual who was a refugee.

**Figure 1:**
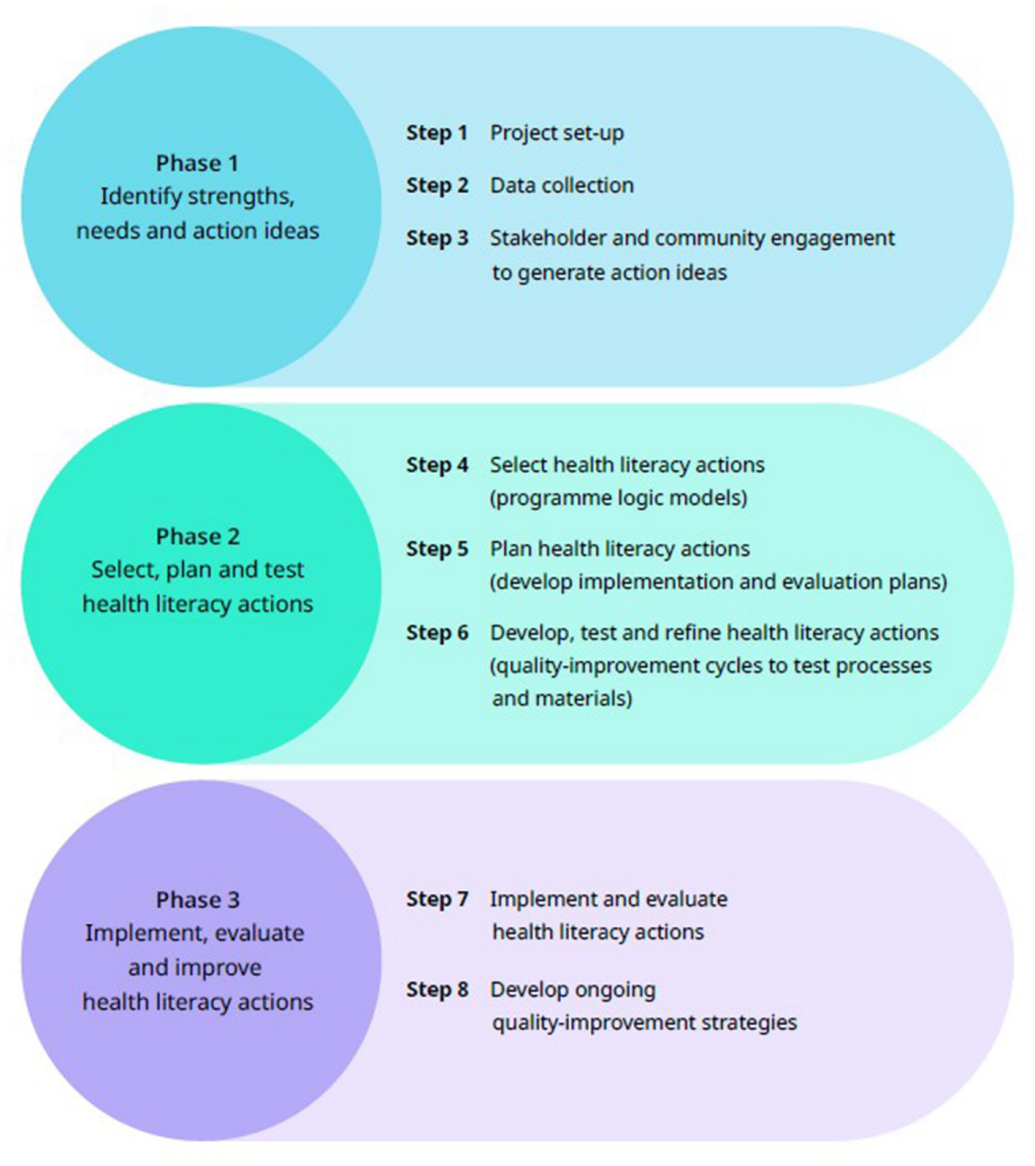
The three phases of the Ophelia process.

Robust research methods that recognize and address health literacy diversity, and consider the contexts of communities, are necessary to develop fit-for-purpose and sustainable solutions to health disparities (46). The Ophelia (Optimizing Health Literacy and Access) process engages community members to help identify and respond to their health literacy strengths and needs. The Ophelia process was developed in Australia (29) and further tested and refined in different contexts and several countries (2, 33, 47–51). It has three phases (Figure 1): Phase 1: needs assessment; Phase 2: co-design and testing of health literacy actions; Phase 3: implementation, evaluation and continuous quality improvement. Typically, the needs assessment in Ophelia Phase 1 uses a multi-dimensional health literacy assessment tool – the Health Literacy Questionnaire (HLQ) – to investigate the diverse health literacy strengths, needs and preferences of groups and communities. Collaboration is undertaken across stakeholder groups, such as community leaders and members, health professionals, managers, and service users to select, test and implement health literacy actions in Phases 2 and 3.

This study protocol describes a health literacy development project that aims to collaboratively identify the health literacy strengths, needs and preferences of a former refugee community living in Melbourne, Australia, and co-design health literacy actions that are culturally and linguistically relevant, meaningful and useful to the community. The Ophelia process will be applied in accordance with the eight Ophelia principles that have been operationalised for this research (Table 2). Health literacy actions will have a strong focus on building the responsiveness of health and community services to support and develop the health literacy of the community. It is expected that the project outcomes will increase the capacity of community members to access, understand, appraise, and use health information and services and enhance their confidence to make informed decisions about their health.

**Table 2:** Principles of the Ophelia process and the application in this study [Adapted from Osborne et al. (32)].

**Principles**	**Description**	**Application in the study**
Focus on outcomes	Focus on improving health and wellbeing outcomes	• We will listen to and respect community voices. • We will give priority to issues of concern to improve the health and wellbeing of former refugee communities. • We aim to equip and empower former refugee communities with the necessary health literacy competencies to increase their capacity to make good health decisions.
Driven by equity	Focus on increasing equity in health outcomes and access to services for people with varying health literacy needs	• The study is designed in a way to ensure the full participation of community leaders and members in all discussions. • The community leaders and members, and other stakeholders will be involved in the decision-making process to ensure ownership and transparency of the study process. • We will emphasize the community values in the development of health actions and interventions that build on their health literacy strengths.
Driven by local wisdom	Prioritize local wisdom, culture, and systems	• We will respect and listen to the lived experience of former refugees. • We will identify and respond to the communities' diverse health literacy needs and preferences to improve their engagement with healthcare services. • We will value their lived experience of migration and settlement.
Diagnosis of local needs	Respond to locally identified health literacy needs	• We will value the lived experiences of community members and other stakeholders. • The study is designed by taking into consideration the context, cultural background, health beliefs and available resources.
Co-design approach	Engage all relevant stakeholders in the co-design and implementation of actions	• In each stage of the Ophelia process, all the stakeholders will be included in the process of developing the study design, suitable data collection tool, action plan, and interventions.
Responsive-ness	Respond to the varying and changing health literacy needs of individuals and communities	• We will recognize the cultural diversity of former refugees and, and their migration history. • We will prioritize their voices and ideas to inform the development of locally and culturally appropriate solutions. • Interventions will be co-designed to increase the community's capacity to understand, access and use health care services and information and enhance their ability to make informed decisions about their health.
Applied across systems	Focus on improvements at and across all levels of the health systems	• The sequence of the study process (e.g., health literacy assessments and workshops) will capture meaningful and evidence-based knowledge over time through the full participation of and engagement with the relevant stakeholders, which will promote changes in different socio-ecological levels.
Sustainable	Focus on achieving sustained improvements through changes to environments, practices, cultures, and policies	• We will work collaboratively with former refugee communities, settlement services, and other community organizations to reinforce sustainable and meaningful interventions through the individual- and community-centered approach. • The collaborative effort is anticipated to advocate for communities needs and preferences. • This study will use culturally and linguistically informed methods, which can be applied in different former refugee population groups.

The setting for this study is a Karen community (i.e., refugees from the country of Myanmar formerly known as Burma) who were identified by AMES Australia, a refugee settlement agency that agreed to form a partnership with the Center for Global Health and Equity, Swinburne University of Technology, including to engage with the Ophelia guiding principles (see Table 2). Consultation then began with the Karen community leaders and members to explore their willingness to participate in a health project. Following endorsement by the Karen community leaders and members and AMES Australia, the project aim was discussed and culturally and linguistically appropriate processes were explored.

## 2. Methods and analysis

### 2.1. Project governance

An Advisory Group was established with community leaders to advise on the aims, purpose and community engagement processes, including recruitment. This group provides advice about relevant tribal and ethnic affiliations and ensures diverse groups will be encouraged to take part.

### 2.2. Setting and participants

The Karen community groups residing in Melbourne mainly originate from rural areas in Burma/ Myanmar. The participants will be recruited by invitation through the Advisory Group, and community and professional networks. Informed consent will be obtained from all participants.

Community members will be invited to participate in semi-structured interviews and/or attend ideas generation and prioritization workshops. The inclusion criteria include people who are:

- A former refugee from Burma/Myanmar;
- Aged 18 years and above; and
- Cognitively able to provide informed consent.

Service providers such as health and social care workers, health practitioners, language support providers, members from the partner organization (AMES Australia), community leaders and clinicians, community nurses, and people who provide direct or indirect services (e.g., policymakers) will be invited to participate in ideas generation and prioritization workshops.

### 2.3. Study design

The study design will be informed by the Ophelia process (see below). The general timeline below provides an approximate schedule for the project.

- July 2021 to 2022 – data collection and analysis (includes interviews, ideas generation workshops, and corresponding analyses)
- July 2022 to 2023 – intervention development, implementation, and evaluation (includes intervention co-design and implementation, and establishing meaningful ongoing monitoring and evaluation strategies).

#### 2.3.1. Phase 1: Identifying the local health literacy strengths, needs and preferences

The Ophelia process is usually conducted using a quantitative data collection method – the Health Literacy Questionnaire (HLQ), which has 44 items in nine scales (four to six items per scale) (30, 33). Cluster analysis is used to analyze the HLQ data, which can be useful to uncover the mechanisms that enable or inhibit groups of people within a population from engaging with health information and services (52). However, quantitative data collection is not appropriate or relevant for every community or culture (26). For example, studies in Aboriginal and Torres Strait Islander communities in Australia identified that yarning methodology (First Nations cultural form of conversation) is an effective and respectful data gathering tool for the Australian First Nations cultures. Yarning nurtures the sharing of knowledge and stories through in-depth discussions (53). This interview technique is a culturally appropriate method of communication transfer to help the Aboriginal and Torres Strait Islander community with chronic disease education and self-management (54). The Karen community in Melbourne has limited English language, as well as limited reading ability in their own language, and there is risk of epistemic injustice if a health literacy measurement instrument that is not meaningful or relevant to their culture is used (26). To minimize the potential for biased data, an open interview technique will be used in this study instead of the HLQ.

To ensure the interview guide captures health literacy dimensions, the Conversational Health Literacy Assessment Tool (CHAT) will be used. The CHAT uses a series of open-ended-questions to facilitate conversations about the ways in which people access, understand, appraise and use health information and services (55, 56). The CHAT consists of five topics, each with 2 questions (total of 10 questions):

1) Supportive professional relationships
2) Supportive personal relationships
3) Health information access and comprehension
4) Current health behaviors
5) Health promotion barriers and support

The CHAT interview guide will be translated into the Karen language by the AMES Karen community liaison officer. The translated CHAT interview guide will be pilot tested with up to 5 community members to check understanding of the questions, issues with the translation, and relevance to the community needs and experiences. Feedback from the community members will be used to inform revisions to the translated questions. Semi-structured interviews will take about 30 min and will be undertaken with the assistance of bilingual workers. Up to 30 community members will be invited to participate in interviews.

Using the needs assessment data, vignettes (evidence-based case studies derived from study data) that portray typical individuals from the target community will be developed. Vignettes are realistic descriptions of profiles of health literacy strengths, needs, and preferences that influence the abilities of groups of people in the community to understand, access, appraise and use health information and services (2). The vignettes will be extensively revised and vetted by participants to ensure they portray the daily lived experiences of the target community members (57, 58).

##### 2.3.1.1. Vignettes development

The health literacy needs assessment data from the interviews and participant demographic data will be used to construct vignettes about community members' experiences and health literacy strengths, needs, and preferences. It is expected that the thematic analysis will yield between 5 and 10 different profiles. Vignettes will be developed for the different health literacy profiles. The social and demographic data will provide narrative information about the contexts in which these experiences may take place. In this way, the vignettes are built to represent the characteristics and challenges of each health literacy profile without representing or revealing specific details about any one individual.

##### 2.3.1.2. Ideas generation workshop

The vignettes will be presented to stakeholders in each workshop with 6 to 10 participants over 2 h. Separate workshops will be held for community members and direct service providers. Language interpretation support will be provided by bilingual workers. In each workshop, participants will be asked 4 key questions based on the vignettes:

1. Do you know people who have had, or have you had, experiences similar to the person in this story [participant is a community member] or Do you see people like this in your community or services? [participant is a service provider]
2. What sorts of problems is this person experiencing in relation to their health?
3. What strategies could be used to help this person?
4. If there were many people like this in your community, what could health services and community organizations do to help?

The Ideas Generation Workshops bring together researchers, community leaders and members, and other stakeholders to participate in discussions about issues facing the community, and to identify what works well and what does not work well for the community. This technique, with a focus on lived experiences embodied in the vignettes, emotes genuine engagement as workshop participants relate to the vignettes (32, 59, 60). Additionally, engaging various stakeholders in the discussion will increase the potential for collaborative efforts to respond to the identified needs using existing resources according to the health literacy strengths, needs and preferences of the community.

#### 2.3.2. Phase 2: Select, plan, develop, and test selected health literacy actions

An action-oriented program logic model and theory will be developed based on the workshop outputs with categorization of short- term, intermediate and long-term outcomes where appropriate (32). This logic model will be co-designed with the key stakeholders to describe the mechanisms for how the generated action ideas are intended to work (61).

#### 2.3.3. Phase 3: Implementation, evaluation and ongoing monitoring of health literacy actions

This phase involves implementation of the chosen health literacy actions from Phase 2, which include improving the local uptake, effectiveness, and sustainability of health actions using quality improvement cycles. AMES Australia will evaluate and examine the intended outcomes (short-term, intermediate, and long-term) of the chosen health actions, and refine the processes to enhance responsiveness and effectiveness, capacity building, and sustainability of the health actions. A post-implementation evaluation tool will be identified and used to evaluate the outcomes (32).

### 2.4. Data analysis

The data will be synthesized in two stages: analysis of the semi-structured interviews and analysis of the data generated from the workshops.

#### 2.4.1. Semi- structured interviews

The narratives from the semi-structured interviews, guided by the CHAT questions, will be coded, themed and analyzed deductively (62–65), whilst also allowing for inductive generation of codes identified in the data. The codes will be categorized and themes will be developed from the categories.

The themes will be used to generate groups of similar health literacy profiles across the interviewed participants. The coded data, within the themes, will be scored according to challenges experienced by participants. A score 3 of 3 means the person experienced fewer challenges; a score of 2 means the person experienced a moderate number of challenges; and a score of 1 means the person had many challenging experiences. The scoring of the challenges will be based on the number and severity of challenges. The severity of the challenges will be determined based on participants' expressions, communication styles, tone of voice and behaviors while responding to the interview questions. Some participants may not give any responses that are relevant to the identified theme, and these will be categorized as “no response”. Scores for each theme will be summed. The total scores will be grouped from the highest scores to the lowest scores. Higher scores suggest potential strengths, and lower scores indicate potential challenges. The demographic data will be linked to each individual to identify demographic differences in terms of education, age, and gender. The health literacy profile of the interviewed community members will be used to develop vignettes.

#### 2.4.2. Ideas generation workshop

In each workshop participants will discuss up to 4 vignettes. The ideas will be grouped into 3 categories of health literacy actions: (1) actions related to what community members can do (e.g., increasing confidence of individuals in using their knowledge and skills of local culture, beliefs, resources, and environment); (2) actions related to what community or local health organizations can do (e.g., understanding local wisdom of the community, providing culturally sensitive services); and (3) policy level actions (e.g., actions that influence organizational policy and decision making processes) (29). Data analysis will be led by one researcher with iterative review and checking of congruency of codes by other members from the team including the community leaders and AMES Australia staff.

Following the thematic analysis of the health literacy actions, a prioritization workshop will be conducted with key stakeholders, including community members and leaders and AMES Australia staff. A health literacy development and implementation plan will be developed.

## 3. Discussion

Health service organizations and settlement agencies who work closely with former refugees experience challenges in identifying, understanding, and responding in culturally responsive ways to the diverse health literacy needs of this cohort. Given that substantial health disparities in former refugee communities are frequently observed (23), new ways to support communities and health authorities to understand the health needs and to take action are warranted. Simply providing health information in different languages for these communities is not sufficient to enhance their engagement with the health services, reduce health inequities, and improve their health outcomes (66–68).

This protocol details an adaptation of the Ophelia process such that it can accelerate a settlement agency's engagement with their community and develop an in depth understanding about how to best generate and implement health literacy development actions and programs that are locally relevant and implementable. Importantly, Ophelia provides an authentic process for engagement and co-design with diverse stakeholders. The Ophelia process has been successfully adapted to fit many projects in different countries around the world (32) and previous Ophelia protocols for studies in different contexts have been published (29, 69, 70) including a study about refugees in Portugal (2). This study will be conducted in accordance with the eight principles of the Ophelia process (Table 2) which may mean that during the co-design phases of the project, some protocol changes may be necessary to suit the cultural and linguistic needs of the community, available resources, and other contextual circumstances.

Potential limitations to this study protocol include inadequate time for consultation and code sign with the agency and community members, limited reach into the full range of refugee groups, and reluctance of refugee groups to express any concerns they may have in their new host country. The governance of the Advisory Group and adherence to the Ophelia principles are, however, likely to mitigate these potential limitations.

The Ophelia process recommends application of a formal multidimensional questionnaire [i.e., the Health Literacy Questionnaire (30)]. However, this type of tool, whether administered in written or oral form, may miss key health literacy elements of the refugee settlement experience, and may be an unacceptable burden to people who are illiterate in the own language, or come from an oral language tradition (26). Consequently, this protocol includes a semi-structured qualitative interview using the CHAT. If successful, this protocol will increase the reach and impact of Ophelia into wider settings, including among groups often not authentically included in research and program development activities. The development and continuous improvement of equitable healthcare services require responsiveness to the local nuances of a community, development of bespoke or tailored health literacy actions, and careful evaluation of the acceptability, uptake and impact of public health responses (32).

## 4. Conclusion

This study will support an organization to understand and respond to the factors that affect a community's ability to understand, access, appraise, and use health information and services to make informed decisions about their health. This protocol applies authentic co-design to develop locally appropriate interventions based on diverse stakeholders' experiences and identified needs. The outcomes of this study are anticipated to be useful for various community-based organizations and policy makers to reduce health disparities in former refugee and other communities that experience vulnerability and marginalization and to create enabling environments that enhance meaningful engagement with and equitable access to health information and services.

## Ethics statement

The studies involving human participants were reviewed and approved by the Swinburne University of Technology Human Research Ethics Committee (Ref: 20214120-5868). Written informed consent for participation was not required for this study in accordance with the national legislation and the institutional requirements.

## Author contributions

ZJ, SE, MH, and RHO contributed to the conceptualization and development of this research design. ZJ drafted the manuscript. All the listed authors reviewed and provided constructive feedback to all manuscript sections and approved the final manuscript.
